# Chronic wound microbiome colonization on mouse model following cryogenic preservation

**DOI:** 10.1371/journal.pone.0221565

**Published:** 2019-08-23

**Authors:** Craig D. Tipton, Nicholas E. Sanford, Jake A. Everett, Rebecca A. Gabrilska, Randall D. Wolcott, Kendra P. Rumbaugh, Caleb D. Phillips

**Affiliations:** 1 Department of Biological Sciences, Texas Tech University, Lubbock, Texas, United States of America; 2 RTL Genomics, Research and Testing Laboratories, Lubbock, Texas, United States of America; 3 Southwest Regional Wound Care Center, Lubbock, Texas, United States of America; 4 Department of Surgery, Texas Tech University Health Sciences Center, Lubbock, Texas, United States of America; 5 Natural Science Research Laboratory, Texas Tech University, Lubbock, Texas, United States of America; University of Illinois, UNITED STATES

## Abstract

Chronic wound infections are increasingly recognized to be dynamic and polymicrobial in nature, necessitating the development of wound models which reflect the complexities of infection in a non-healing wound. Wound slough isolated from human chronic wounds and transferred to mice was recently shown to create polymicrobial infection in mice, and there is potential this tool may be improved by cryogenic preservation. The purpose of this study was to investigate the application of cryogenic preservation to transferring polymicrobial communities, specifically by quantifying the effects of cryopreservation and wound microbiome transplantation. Slough from an established murine polymicrobial surgical excision model and five patients were subjected to three preservation strategies: refrigeration until infection, freezing in liquid nitrogen, or freezing in liquid nitrogen with glycerol solution prior to infection in individual mice. Four days following inoculation onto mice, wound microbiota were quantified using either culture isolation or by 16s rRNA gene community profiling and quantitative PCR. Cryogenic preservation did not significantly reduce bacterial viability. Reestablished microbial communities were significantly associated with patient of origin as well as host context (i.e., originally preserved from a patient versus mouse infection). Whereas preservation treatment did not significantly shape community composition, the transfers of microbiomes from human to mouse were characterized by reduced diversity and compositional changes. These findings indicated that changes should be expected to occur to community structure after colonization, and that compositional change is likely due to the rapid change in infection context as opposed to preservation strategy. Furthermore, species that were present in higher relative abundance in wound inoculate were more likely to colonize subsequent wounds, and wound inoculate with higher bacterial load established wound communities that were more compositionally similar. Results inform expectations for the complementation of chronic wound in vivo modeling with cryogenic preservation archives.

## Introduction

Chronic wounds and associated treatment continues to be a burden on patients and the healthcare system, frequently resulting in high financial costs, recalcitrant infections, and devastating outcomes such as amputation [1, 2]. Wound etiologies can vary [3], but there is general agreement that infection by various microbial species directly interfere with the normal healing process and cause wounds to persist [4–9]. The treatment of wound infections is complicated by biofilm formation, which is characterized by enhanced resistance to host immune responses and antimicrobial perturbation [10, 11]. Furthermore, biofilm-associated cells display altered phenotypes as compared to planktonic cells, evidenced by observed physiological responses and complex synergistic interactions [10–15]. Wound microbiomes are usually polymicrobial in nature, with diversity in the community composition of bacterial and fungal species [5, 7, 13, 16, 17]. The importance of addressing the multitude of species present within a wound is supported by studies demonstrating significant temporal variation in wound community structure and the ability for relatively low abundant species to later dominate the wound microbiome [17, 18]. Approaching the care and research of chronic wound infections as dynamic polymicrobial biofilms is thus necessary to understand the complex dynamics underlying chronic wound microbiomes.

In practice, the investigation of chronic wounds and polymicrobial infections is met with various challenges. While numerous insights have been made from clinical studies [7, 18–20] and *in vitro* systems [13–15, 21], *in vivo* studies are valuable for modeling infection in context with a host, allowing experimental designs which would otherwise be clinically unethical or impractical [10, 13]. Several chronic wound *in vivo* models have been employed to study infection dynamics including various murine models [4, 22–24], the rabbit ear model [9, 16, 25], the porcine model [26], and an *ex vivo* skin explant model [27]. The different model systems have been proposed to offer different advantages depending on the question [10, 13], but a common theme among these studies and others is infection of the model organism with planktonic-favoring cultures, often with much lower diversity than is seen in patient wounds [5, 9, 10, 13, 14, 16, 22–27]. There are purposes for working from stocks of carefully archived strains, but a limitation is that doing so may not accurately reflect the dynamics of chronic wound biofilm phenotypes nor their communities [10, 13]. The ability of patient-isolated samples to re-establish polymicrobial biofilm communities in a murine model was recently investigated [4] where it was reported that 81% of wound isolates were able to reproduce polymicrobial infections between mouse and patient, though the compositional similarity between the microbiota of these samples was not thoroughly evaluated in that work. Furthermore, a technical challenge associated with studies integrating clinical and live animal approaches is the ability to isolate wound slough at the clinic, transport samples, and then perform infection procedures in a timely manner [4].

The archival of question-relevant tissue enables experimental designs that address complex biological patterns, such as those that are temporal in nature or otherwise difficult to survey [28, 29]. In particular, cryogenic biobanking of tissues enhances genomic, transcriptomic, and metagenomic research by preserving sample quality through time [29, 30]. Cryopreservation at -80°C has been demonstrated to maintain bacterial cell preparation viability up to 10 years and storage in vapor phase liquid nitrogen (< -139°C) up to 30 years [31]. The benefit of archiving samples at ultra-cold temperatures is that molecular activity is minimal below -139°C, thus reducing chemical reactions and promoting cellular integrity [31]. Cell wall structure has been indicated to affect cell viability, with Gram-negative bacteria being less viable than Gram-positive species after freezing [31]. To counteract this selective effect, the use of cryoprotectants (e.g., glycerol) or initial ultra-cooling by liquid nitrogen has been advised [31, 32]. However, the above referenced work was based on monoclonal cultures, and it is currently unknown how polymicrobial communities respond to freezing. Thus, the aim of the current study was to investigate microbiome community viability following cryogenic preservation, as well as to characterize sources of variation in microbiome community composition following re-establishment in a murine model after cryogenic archival by using a pre-established wound community in mice and communities from patient wounds.

## Materials and methods

### Ethics Statement

This study was carried out in strict accordance with the recommendations in the Guide for the Care and Use of Laboratory Animals of the National Institutes of Health. The protocol was approved by the Institutional Animal Care and Use Committee of Texas Tech University Health Sciences Center (protocol number 07044). Mice were anesthetized using 0.02 ml per gram weight of Nembutal (pentobarbital sodium, 5 mg/ml) and euthanized with 150 μl of Fatal-Plus solution (pentobarbital sodium, 390 mg/ml). Human study protocol was executed after obtaining informed written consent from adult patients for study as approved under Western Institutional Review Board protocol (Protocol number: 20111320).

### Community preparation, sampling, and processing

Bacterial strains were grown to establish a polymicrobial *in vitro* wound-like community. *Staphylococcus aureus* JE2 (PMID 27582729), *Pseudomonas aeruginosa* PAO1 (ATCC BAA-47), and *Corynebacterium striatum* (ATCC 6940) were grown individually overnight at 37°C in Brain Heart Infusion broth shaken at 180 rpm in atmospheric oxygen levels. *Finegoldia magna* (ATCC 29328) was grown for 48 hours at 37°C in reduced Clostridium broth in 8% H_2_, 10% CO_2_, balanced N_2_. *In vitro* polymicrobial wound-like communities [33, 34] were created in 6 x 50 mm glass tubes containing 460 μl of Bolton’s broth supplemented with 5% laked horse blood and 50% bovine plasma, then subsequently inoculated with 5 μl of each culture normalized to 1x10^6^ colony forming units (CFU). Communities were grown stationary in atmospheric oxygen levels for 48 hours, then weighed and homogenized in 1 ml PBS with 2.4mm metal beads at 4 m/s for 30 s, serially diluted and selectively enumerated for CFU on Pseudomonas isolation agar for *P*. *aeruginosa*, mannitol salt agar for *S*. *aureus*, and Hoyle’s agar with 20 μl /ml fosfomycin for *C*. *striatum* grown 24–48 hours at 37°C in atmospheric oxygen; anaerobic basal agar for *F*. *magna* was used in anaerobic conditions grown 48 hours and differentiated by colony morphology. The synthesized wound-like community is subsequently referred to as generation zero, the first transplanted communities in mice as generation one, and communities established on a secondary mouse following preservation treatment referred to as generation two.

#### Mouse wound samples

To evaluate viability of communities after cryopreservation and subsequent transplantation in mice, murine wounds were created using a known, pre-established community. Samples were created as previously described [22] by using a polymicrobial full excision wound model. Briefly, three Swiss Webster female mice were fully anaesthetized, then hair on backs shaved and removed with depilatory cream. Analgesic 1% 0.05 ml lidocaine was injected subcutaneously at the site of excision prior to fully excising a 1.5 cm x 1.5 cm area of all skin layers. Open sterile wounds were inoculated with an *in vitro* wound-like community (100mg) and covered with an OPSITE IV3000 semi-permeable polyurethane dressing (Smith & Nephew, London, United Kingdom). Infection proceeded for 4 days, then mice were euthanized and six equisized portions of wound tissue were collected, manually mixed by curette, and immediately subsampled for cryopreservation treatments or CFU. For microbial CFU, wound tissue was weighed and homogenized in 1 ml PBS with 2.4 mm metal beads at 4 m/s for 60 s, serially diluted, and isolated and enumerated as described above.

#### Patient wound samples

Patient samples were collected at the Southwest Regional Wound Care Center in Lubbock, TX. Adult patients were selected on the basis of having a lower extremity wound that had persisted for at least a month and showed signs of infection, visible by wound exudate. Sampling included five patients whose wound slough was used for mouse infection, as well as an additional 20 patient samples that were used for viability assays. For each patient, wound slough was collected during aggressive debridement as part of their standard of care in which removed slough was manually mixed by curette, and then immediately subsampled for downstream preservation treatment.

#### Cryopreservation treatments

To study the effect of preservation method on the ability of microbiome communities to re-establish on a mouse model, slough was subsampled into three separate approximately 50 mg samples: One subsample was frozen directly in liquid nitrogen, the second was first mixed by brief vortexing in 100 μl 1:1 glycerol:PBS prior to freezing in liquid nitrogen, while the third was refrigerated at 4°C prior to inoculation on mice. Mouse-mouse transplant communities included an additional slough sample to immediately inoculate mice without preservation. Slough subsampling also included a 15 mg sample that was either immediately frozen in liquid nitrogen and later used to characterize wound microbiome characteristics at time of collection using 16s rRNA community profiling and bacterial load quantification by qPCR, or a 50mg sample used for CFU quantification in mouse-mouse transplants. Patient samples were transported by dewar or on ice packs to the Texas Tech University Health Sciences Center and returned to refrigeration at 4°C or a freezer at -80°C.

### Viability experiments

#### Transplants into mice

Following collection and preservation of wound slough, 30 adult female Swiss Webster mice (Charles River Laboratory, Wilmington, MA) were anaesthetized and wounded following previously reported procedures [22]. Frozen slough samples were briefly thawed at room temperature. To produce generation two communities, mice were then infected with experimentally preserved slough samples which were directly inoculated onto open sterile mouse wounds. Wounds were then covered by OPSITE IV3000 semi-permeable polyurethane dressing (Smith & Nephew, London, United Kingdom). Mice were co-housed according to treatment and, for patient-mouse wound transplants, a control mouse that was wounded but not inoculated with wound slough was included in each cage. At four days post inoculation, mice were euthanized, and wound beds were excised for CFU (mouse-mouse) as previously described or 16s rRNA gene community profiling and bacterial load quantification by qPCR (patient-mouse). CFU counts were standardized by mass, log-transformed, and then a nested three-way ANOVA was used to evaluate differences in viability after transplant.

#### PMA-qPCR of patient samples

To determine viability upon collection of samples, clinically isolated patient wound slough was portioned into six different 40 mg aliquots, two for each of the preservation treatments. Refrigerated samples were processed in under 24 hours. Samples that were frozen immediately in liquid nitrogen or stored in 100 ul 50:50 glycerol:PBS before freezing, were then stored at -80°C for 3–14 days until analysis. All samples were processed by thawing, adding 500 μl PBS (or 400 μl to glycerol samples), and a steel bead (440c, 0.187” diameter) before disrupting at 25 Hz for 30 s on a Qiagen Tissue Lyser II (Qiagen). For each preservation treatment, one sample was left untreated and the other was treated with propidium monoazide (PMA), which is a photo-reactive molecule capable of entering compromised cells and binding with DNA [35]. For PMA-treated samples, 1.25 μl PMA was added and briefly vortexed. Tubes were then incubated at room temperature in the dark for 20 minutes on a rotating shaker set at 85 RPM. After incubation, PMA was irreversibly bound to extracellular DNA by exposing the tubes to PMA-Lite (Biotium, Fremont, CA) for 15 minutes.

DNA from PMA treated and untreated samples was extracted by Roche High Pure PCR Template Preparation kit following manufacture protocol (Roche, Pleasonton, CA). Cell lysis step included the use of 0.5 mm zirconium oxide beads (Next Science, Jacksonville, FL). Cells were lysed on Qiagen Tissue Lyser II for 5 minutes at 30 Hz. Quantitative PCR was carried out on Roche Light Cycler 480 (Roche Life Sciences, Indianapolis, IN) using the Quanta Bioscience PerfCta PCR toughmix in 12.5 μl with the following thermal profile: 1 cycle of 50°C for 2 min, 1 cycle of 95°C for 10 min, followed by 35 cycles of 95°C for 15 sec, 60°C for 1 min, and a final cycle at 40°C for 30 sec. Primers used were as follows: Forward, 5’-CCATGAAGTCGGAATCGCTAG-3’; Reverse, 5’-GCTTGACGGGCGGTGT-3’; and Probe, 5’-TACAAGGCCCGGGAACGTATTCACCG-3’. Positive cycle threshold (Ct) results were scored if detected at or below 30 cycles, and negative if not detected by 30 cycles. To assess the effects between preservation treatments, the Ct values for PMA treatments were subtracted from the untreated sample Ct values. Two-way ANOVA was used to determine whether there was differential viability among preservation treatments, while controlling for variation between individual patients.

### Patient microbiome analysis

To assess microbiota from the patient-mouse transplants, sequencing and qPCR was conducted by RTLGenomics (Lubbock, TX). In brief, DNA was extracted from tissue using a Qiagen TissueLyser II (Qiagen, Valencia, CA) and High Pure PCR Temple Preparation Kits (Roche). Sample amplification for sequencing was conducted using primers encompassing variable regions 1 through 3 of the 16s rRNA gene as previously described [36]. Sequencing was conducted on the Illumina MiSeq platform (Illumina, San Diego, CA) using manufacture protocol and targeting a minimum depth of 10,000 taxonomically classified reads per sample. Raw sequence data were deposited with the NCBI (Accession number: SRA676483). Raw reads were quality filtered, clustered into operational taxonomic units and taxonomically assigned using a 16s database developed in-house at MicroGen DX (Lubbock, TX) as previously described [5, 18]. Microbiome diversity is subsequently reported at the species-level, but due to the unknown misclassification rate, species epithets should be considered as tentative.

For 16s qPCR, DNA was amplified using a Light Cycler 480 (Roche Life Sciences) with the Roche LC480 master mix (Roche Life Sciences). Reactions were prepared using manufacture protocols in 10 μl volumes with the thermal profile and primers referenced above for cell viability analysis.

#### Statistical analysis

Statistical analyses were conducted in R version 3.3.1 [37]. Before each use of a parametric test, assumptions of homoscedasticity and normality were tested to determine the appropriate analysis. To assess sequencing effort, rarefaction curves were generated by subsampling community matrices between 100 and 10,000 reads at a step size of 100 reads, and the mean number of observed species of 10 iterations was calculated at each step. The bacterial species community matrix was Hellinger transformed [19] to alleviate compositionality bias inherent in proportional community data. Effects of patient and preservation treatment on microbiome alpha (species richness) and beta diversity (Bray-Curtis Community Dissimilarity [38]) were assessed using nested ANOVA and PERMANOVA (ADONIS [39]), respectively. T-tests were used to confirm that cage-mates had no significant effect on each other’s wound microbiome by partitioning Bray-Curtis distances between control mice to cage-mates and control mice to mice not cohabitating during study. To compare diversity patterns among samples, non-metric multidimensional scaling (NMDS) ordination was used to cluster microbial communities based on Bray-Curtis dissimilarity [40].

ANOVA was used to determine effects of patient and treatment on observed bacterial load in mice. Following significant results, Tukey’s Honest Significant Difference (HSD) post-hoc testing was applied to determine which comparisons contributed to overall statistical significance. To evaluate if bacterial load could serve as an indicator for how similar a patient’s original microbiota was to the communities established on the mouse model, samples were grouped by the bacterial load of their isolated patient sample. Initial load groups were defined as being “<LOD” (Not detected in qPCR assay, n = 2 patients), “Med” (Ct value between range of 25–28, n = 2 patients), and “High” (Ct value equal to 16.59, n = 1 patient). The three group conditions noted encapsulated all 5 original patient wounds. The relationship between bacterial load group classification and patient-specific Bray-Curtis distances from patient to mouse were evaluated by a Kruskal-Wallis test. Next, the relationship between species richness and bacterial load was evaluated by log transformation and Analysis of Covariance (ANCOVA), incorporating the variable ‘patient’ as a covariate in a linear regression model.

Bacterial persistence was investigated by separating bacterial species that were present in the first patient wound into groups based on whether they established, or failed to establish, in at least one mouse wound. The relative abundances of these species prior to mouse infection were then compared by Welch’s non-parametric t-test. The effects of Gram’s stain and aerotolerance on species persistence through colonization were each assessed by chi-square test.

## Results

**Fig 1:**
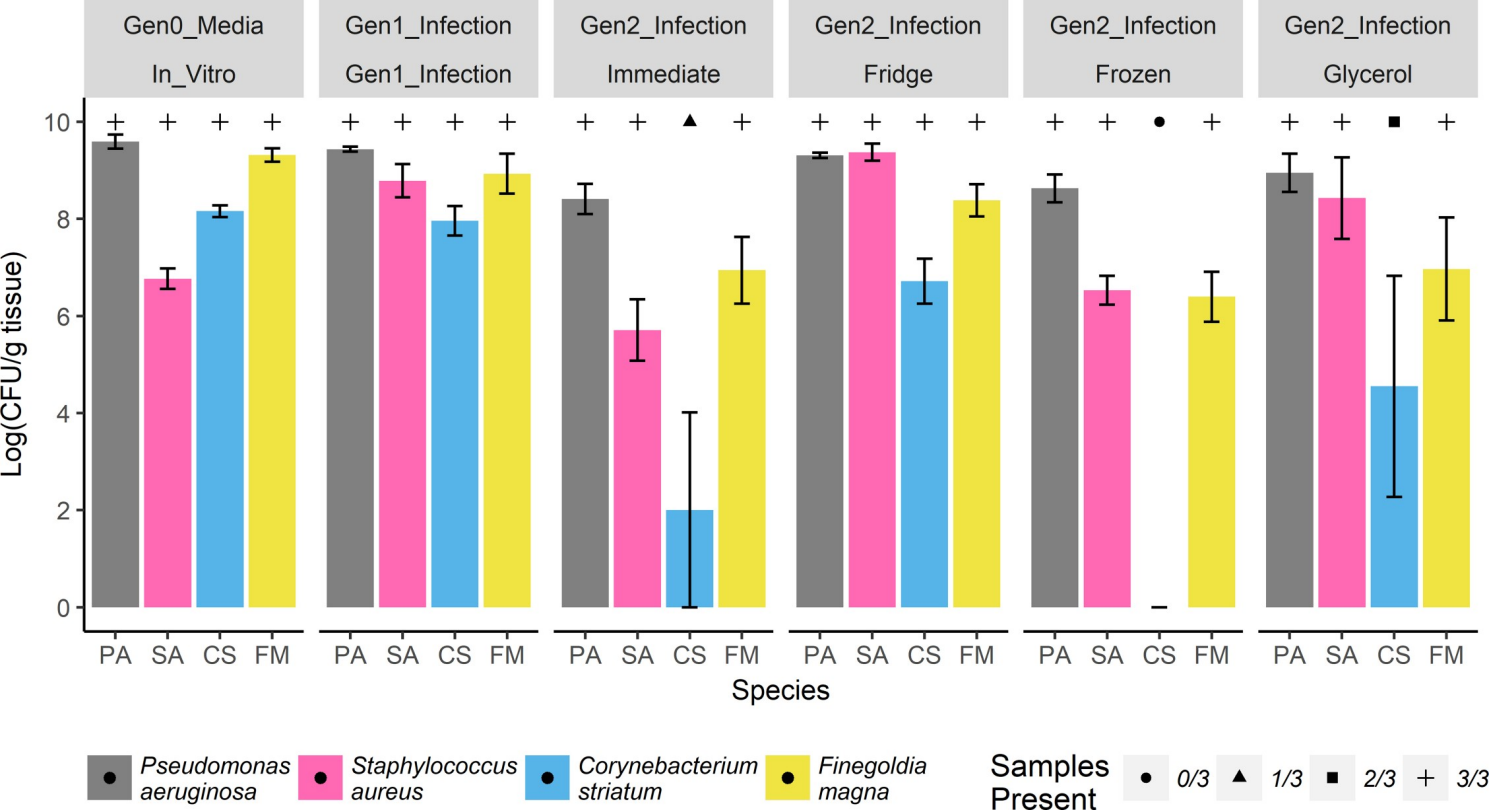
Polymicrobial samples are viable through freezing. Bar plot of species abundances across three mouse replicates (Mean +/- Standard Error). Samples are grouped by community generation and preservation treatment. Generation zero denotes community synthesis in wound-like media, generation one being infection (without treatment) in a mouse, and generation two being infection onto subsequent mice after wound slough extracted from generation one mice received each preservation strategy. Shapes above individual bars indicate the number of mice which a particular species was detected by CFU count.

Bacterial viability in response to the preservation strategy was assessed in each of three experiments, with the mouse-mouse transplant experiment specifically investigating the ability of bacteria to be cultured following cryogenesis in a multi-species model. In the mouse-mouse transplant experiment, three of the four bacterial species chosen to synthesize wound-like communities appeared in every subsequent wound through treatment as demonstrated by CFU determination (Fig 1). A nested ANOVA model was used to assess the relative influence of community transfer events, preservation strategy, and the contribution of specific species to CFU abundance. Overall abundance was significantly less in generation two infections (F = 10.47; df = 2, 62; p < 0.001; R^2^ = 0.126) and preservation treatment was detected to have a significant effect as nested within generation two (F = 19.56; df = 3, 62; p < 0.001; R^2^ = 0.126). Specifically, generation two abundances were reduced in communities resulting from immediate transfer and from cryogenic freezing without glycerol (p < 0.05), while those from refrigerated and glycerol treated frozen aliquots were statistically indistinguishable from synthetic and generation one infections (p > 0.90, Fig 1). Individual species contributions were found to vary significantly (F = 19.56; df = 3; p < 0.001; R2 = 0.352), with *C*. *striatum* exhibiting the greatest reduction in abundance and being the only species which was not detected in some generation two mice (Fig 1). Being derived from the same synthetic *in vitro* community explained non-significant variation (F = 0.052; df = 1, 62; p = 0.820; R^2^ < 0.001).

**Fig 2:**
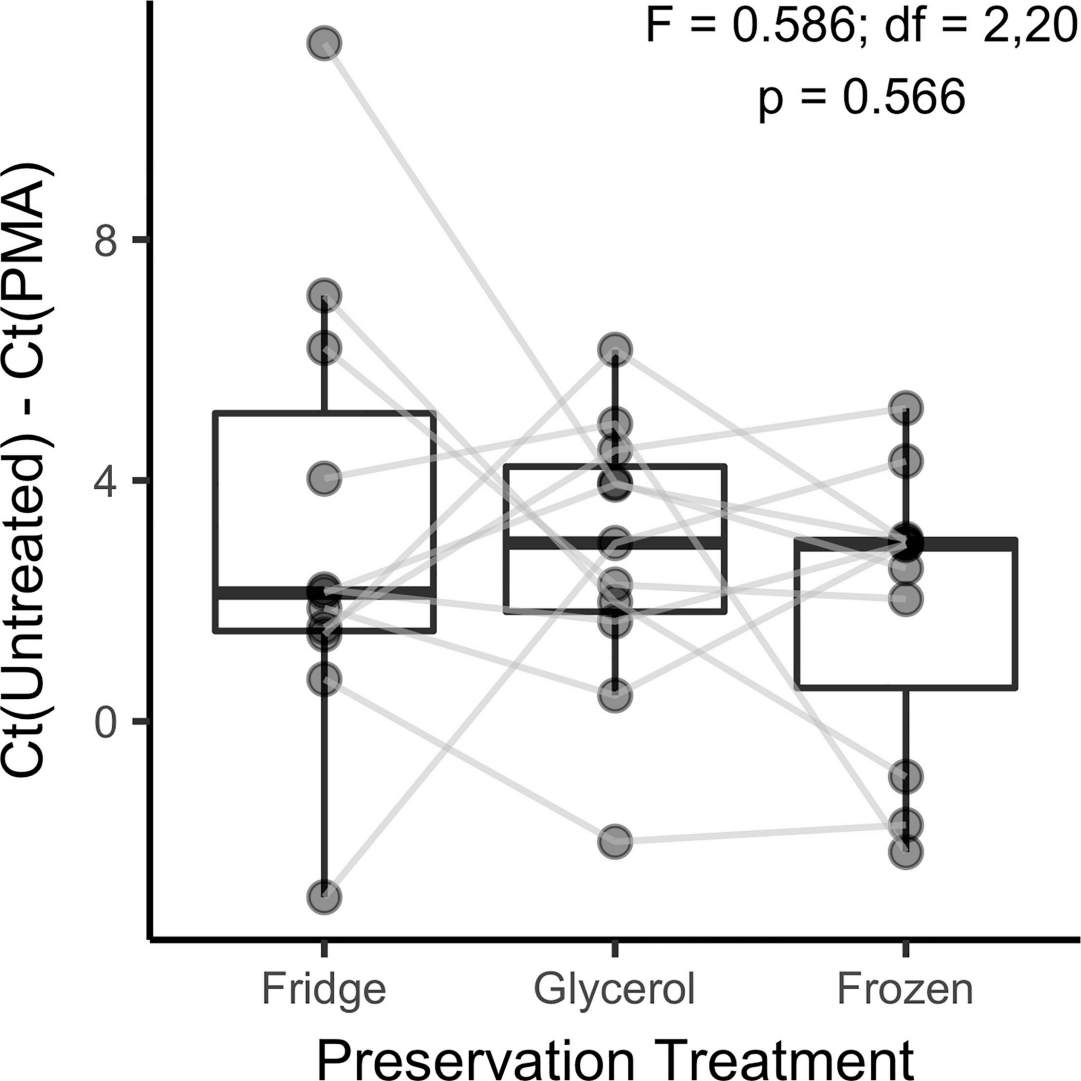
Cell viability was not reduced by freezing. Analysis of the effect of preservation treatments on cell viability from slough from 11 patients assessed by PMA-qPCR. The difference in non-treated and PMA-treated samples are displayed on the y-axis and gray lines connect measurements taken from the same patient wound.

A second experiment to test bacterial cell viability through cryogenesis involved sampling 20 patients and dividing their slough for experimentation via PMA-qPCR. Quantitative PCR scoring was successful in eleven of these patients (all subsample assays were negative for the other nine wounds indicating bacterial burden below the limit of detection at 30 qPCR cycles). For the eleven positive wounds, testing by two-way ANOVA did not indicate that preservation type (F = 0.586; df = 2, 20; p = 0.566) or patient (F = 1.063; df = 10, 20; p = 0.432) significantly influenced cell viability (Fig 2).

### Microbiome analysis

**Fig 3:**
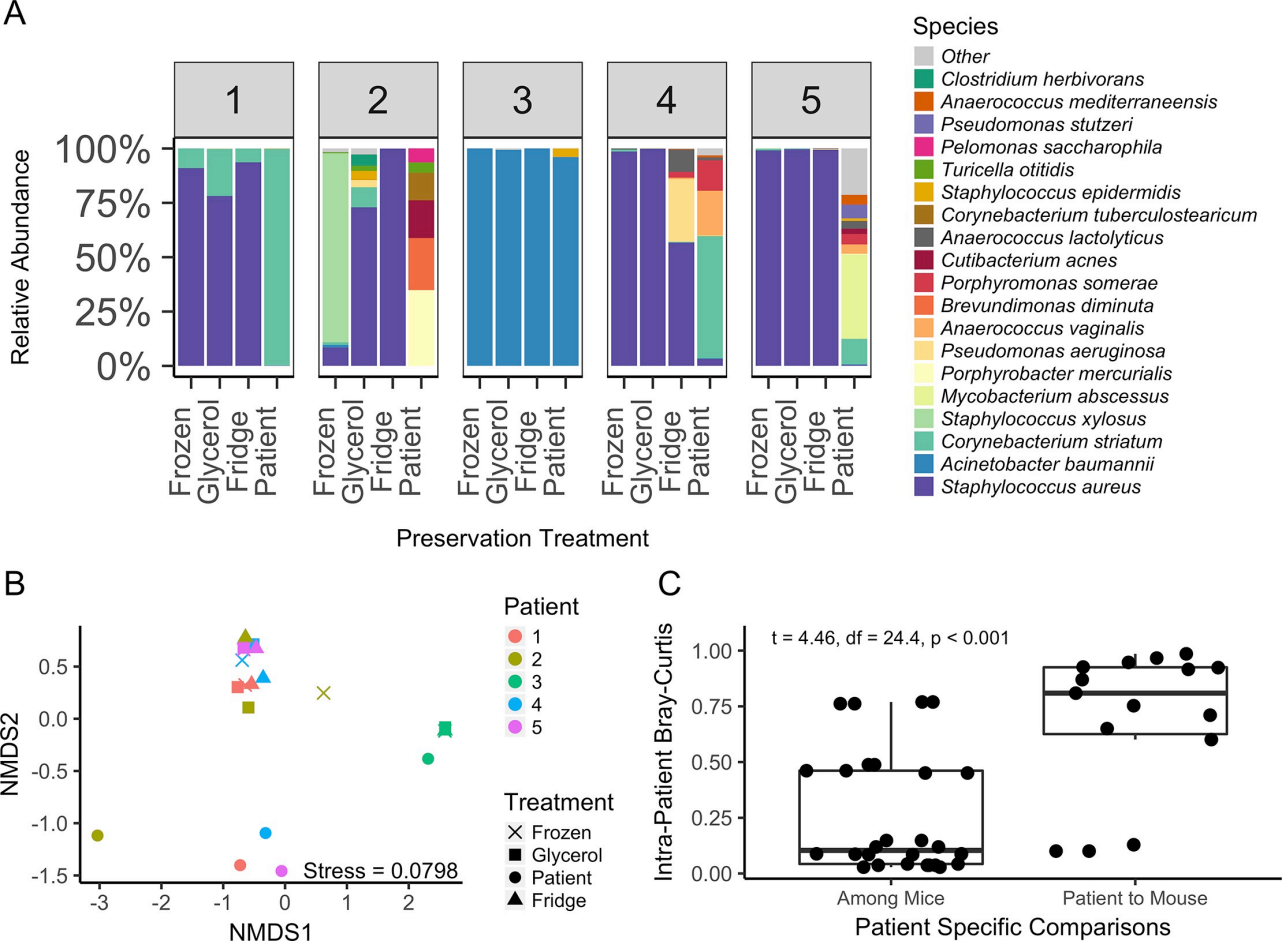
Compositional variation among wound microbiomes is influenced by host context and patient origin. Briefly, five patient wounds were sampled at the clinic. Their wound isolates were divided into three different preservation strategies per patient sample (e.g. ‘Fridge’, ‘Glycerol’, ‘Frozen’) and a fourth sample aliquot was sent directly for sequencing (‘Patient’). After preservation, sample aliquots were then used to infect surgical excision mouse wound models and sampled again at day four for sequencing. A) Relative abundance bar plot of the overall 19 most prevalent species with wounds grouped by patient isolate and each bar representing an individual mouse receiving the specified treatment. B) Compositional variation in wound microbiomes summarized using Bray-Curtis dissimilarities and non-metric multidimensional scaling (NMDS). C) Patient specific mouse-mouse and patient-mouse comparisons of microbiome compositional similarity. On the y-axis a value of 0 indicates identical communities while 1 indicates communities with no species in common.

Rarefaction survey indicated sequencing effort was sufficient in characterizing wound microbiota (S1 Fig), although some samples had fewer classified reads despite extensive sequencing effort (many reads for these samples were identified as mouse). A t-test comparison of Bray-Curtis dissimilarity between control mice and cage-mates versus control mice and mice house separately indicated that co-housing did not influence community composition (t = 0.198, df = 43, p = 0.422). The median species richness was 6 across all samples (1^st^ Quartile = 2.5, 3^rd^ Quartile = 10) and 5 after removing control mice (1^st^ Quartile = 2, 3^rd^ Quartile = 7.25). Bacteria are listed in order of decreasing relative abundance with the top five most abundant observed in this study being *Staphylococcus aureus*, *Acinetobacter baumannii*, *Corynebacterium striatum*, *Staphylococcus xylosus*, and *Mycobacterium abscessus* (Fig 3A). Evaluating differences in alpha diversity across samples, nested ANOVA testing yielded non-significant effects of treatment (F = 0.576; df = 2, 12; p = 0.576; R^2^ = 0.047) and patient (F = 1.76; df = 4, 12; p = 0.201; R^2^ = 0.287), but did detect a notable richness decline from patient wounds to mouse wounds after colonization (F = 4.37; df = 1, 12; p = 0.056, R^2^ = 0.178).

Next, effect of patient and treatment type on mouse beta diversity was assessed. NMDS [38] ordination plot was used for variable reduction and to visualize diversity patterns among samples (Fig 3B). Qualitatively, wound samples dominated by *S*. *aureus* cluster together, original wound samples cluster with the exception of patient 3, and samples related to patient 3 clustered together. To quantify these effects, ADONIS was used to detect significant effects based on the patient inoculated from (F = 6.46; df = 4, 12; p < 0.001, R^2^ = 0.541) and significant differences after patient to mouse colonization (F = 6.47; df = 4, 12; p = 0.002; R^2^ = 0.188), but non-significant effects of treatment on microbiota composition (F = 0.468; df = 2, 12; p = 0.900, R^2^ = 0.019). The effect of the patient samples dominated by *Acinetobacter* on test results was assessed by removing Patient 3 samples and repeating the analysis to focus on the *Staphylococcus* cluster. In this analysis neither patient effect (F = 1.18; df = 3, 6; p = 0.195; R^2^ = 0.314) nor treatment effect (F = 0.859; df = 2, 6; p = 0.736, R^2^ = 0.152) remained significant, albeit explaining approximately 31.4% and 15.2% of variation, respectively. Qualitatively, original patient samples appeared consistently more different from their corresponding mice, as compared to among mice receiving the same wound slough (Fig 3A and 3B). To quantify the effect of human or mouse origin of each community Bray-Curtis dissimilarities of each patient and their derived mice were compared by Welch’s t-test and found to be significant (t = 4.46, df = 24.4, p < 0.001; Fig 3C). After determining that the composition of mouse versus patient microbiota was significantly different, whether any preservation strategy yielded mouse communities that were more similar to their respective patients was investigated (i.e., by directly comparing Bray-Curtis dissimilarities of patient:mouse pairs among treatments) and was not significant (F = 0.004; df = 2, 12; p = 0.996; R^2^ < 0.001).

#### Bacterial load as a predictor

**Fig 4:**
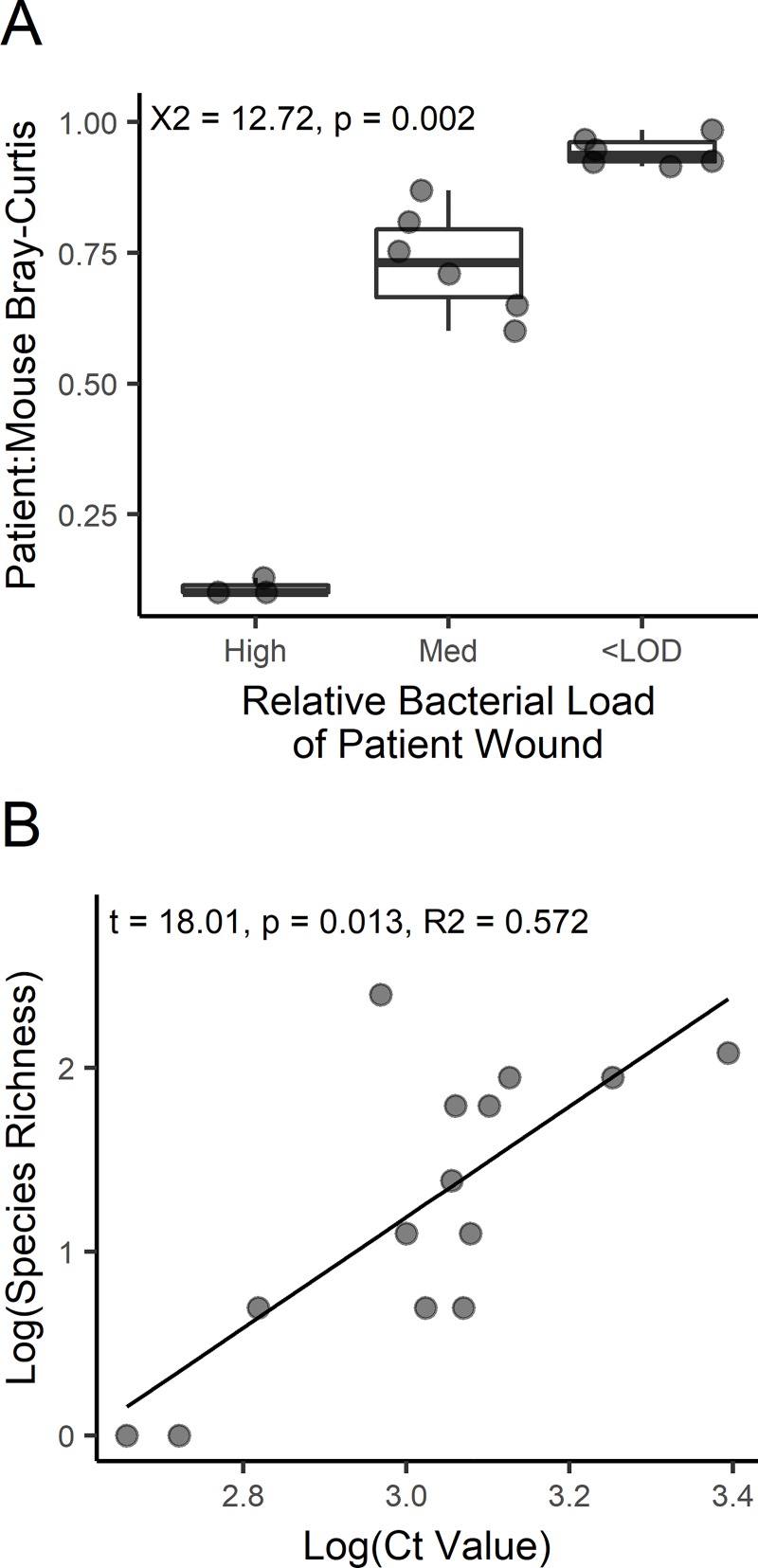
Bioburden and diversity of patient wounds significantly influenced colonization on mice. A) The distribution of microbiome dissimilarity between patient and corresponding mice categorized by bacterial load of patient wounds. B) Regression of log transformed observed species richness and bioburden. Note that a low cycle threshold (Ct) value indicates high bacterial load.

To assess patient and treatment effects on the established mouse wound bacterial load, two-way ANOVA was used to determine significance of patient effect (F = 19.83; df = 4, 7; p < 0.001; R^2^ = 0.912) and non-significance of treatment effect (F = 0.35; df = 2, 9; p = 0.719; R^2^ = 0.008), explaining approximately 91% and 1% of variation, respectively. Using Tukey’s HSD, it was found that mice infected from patient 3 had consistently higher loads when compared to other patient derived mice (adj. p < 0.05), mice from patient 2 had the lowest bacterial loads compared to other mice (adj. p < 0.05), and mice from patients 1, 4, and 5 were all found to possess similar bacterial loads (adj. p > 0.9). The highly significant patient effect indicated that original patient wound bacterial load could predict how well a wound establishes on the mouse model. Using a Kruskal-Wallis test, it was determined that compositional similarity among mouse wounds was non-randomly distributed with respect to bacterial load (X^2^ = 12.17, df = 2, p = 0.002), and patient inoculum with high bacterial loads yielded wounds that were more compositionally similar (Fig 4A). Next, a relationship between bacterial load and observed species richness was investigated using analysis of covariance (ANCOVA) of log transformed data, through which a significant linear relationship was estimated (t = 18.01; df = 1, 4; p = 0.013, R^2^ = 0.572; Fig 4B). Neither the patient (t = 0.548; df = 4,4; p = 0.713; R2 = 0.070) nor interaction (t = 1.773; df = 4,4; p = 0.289; R2 = 0.231) terms were significant in this model.

#### Bacterial persistence

**Fig 5:**
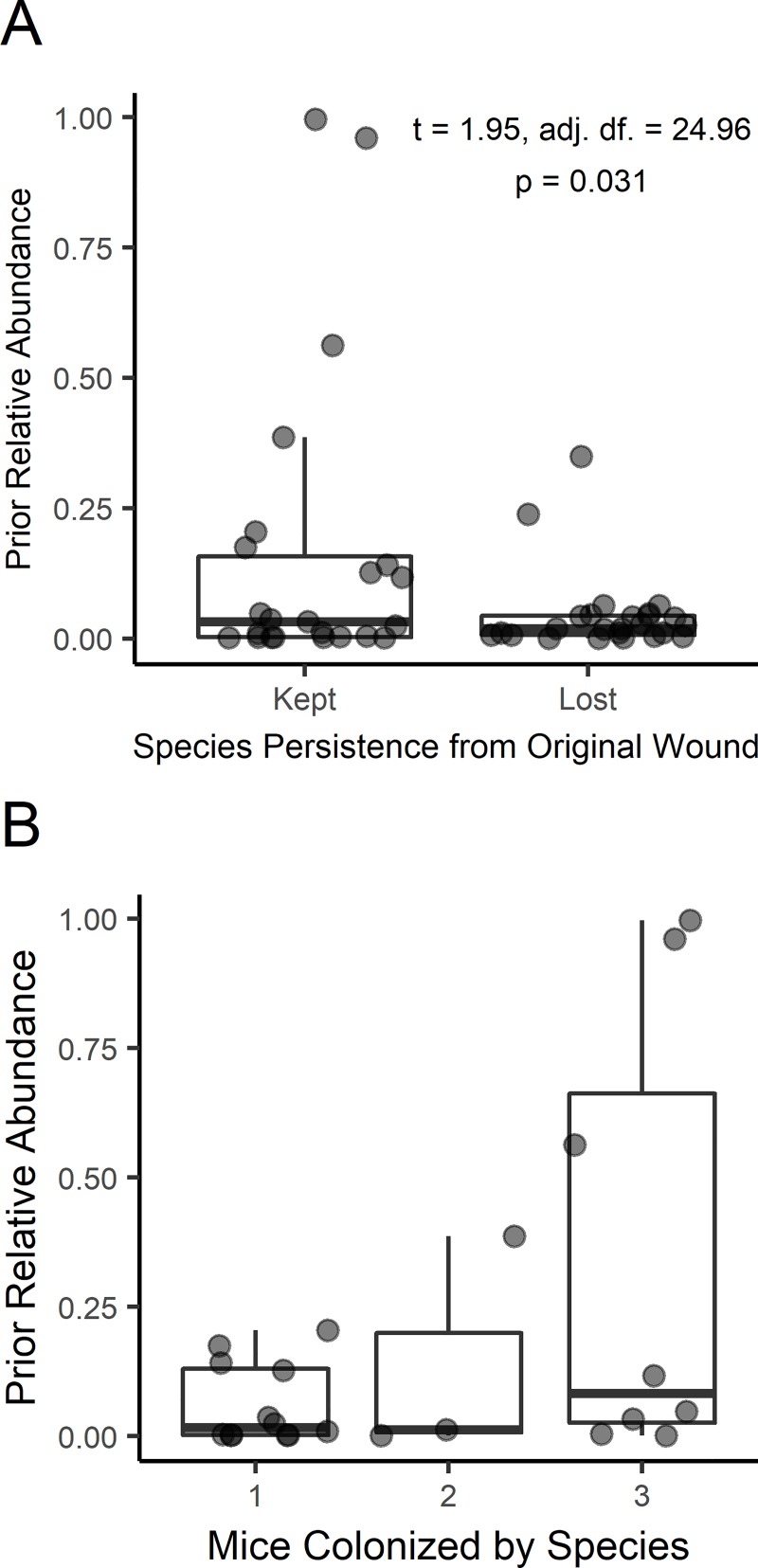
The relative abundance of bacterial species in patient inoculate influenced establishment on mouse wounds. The effect of species relative abundance observed in patient microbiomes on subsequent detection in mouse wounds was assessed by A) grouping species as detected or undetected, and by B) the number of mice per patient in which a given bacterial species was observed. Each point represents one relative abundance value of a single species in a single patient before transfer into mice.

Next, the prior relative abundance of species in the inoculum was investigated for determining which species persisted from the patient to the mouse model. The influence of prior relative abundance on subsequent species establishment (S1 Table, Fig 5A) was assessed using Welch’s t-test and found to be significant (t = 1.95, adj. df. = 24.96, p = 0.031). Bacterial persistence was further investigated by assessing how often a species was observed in each of the three replicate mice (Fig 5B). As there were only two instances where a microbe persisted in two of three wounds, statistical analysis was conducted between one and three count groups. Using Welch’s t-test, there was a notable effect of prior relative abundance on the number of subsequent wounds that a particular species was detected in (t = 1.76, adj. df. = 7.48, p = 0.059). The relationship between prior relative abundance and colonization success was specifically addressed for *S*. *aureus* by regressing prior relative abundance on colonized relative abundance, through which a non-significant relationship was observed (t = 1.18, df = 13, p = 0.26, R^2^ = 0.10). The mean relative abundance of *S*. *aureus* in mice was 66.5% (n = 15 mice) despite a prior relative abundance mean of 0.8% (n = 5 patients). To determine the effect of phenotypic influence on species persistence from patient to mouse, chi-square testing indicated non-significant effects for Gram designation (X^2^ = 0.697, df = 1, p = 0.404) and aerotolerance (X^2^ = 1.56, df = 2, p = 0.458).

## Discussion

This study assessed how cryogenic preservation influenced the ability of polymicrobial communities to reestablish on mouse wound models. During the mouse to mouse transplant experiments, *C*. *striatum* was notably less successful in re-colonizing from a pre-established wound compared to *P*. *aeruginosa*, *S*. *aureus*, and *F*. *magna* (Fig 1). Given that *C*. *striatum* was not detected at all in frozen samples lacking glycerol may suggest a poor response to cryopreservation, though *C*. *striatum* was also absent from two of three infections resulting from immediate slough transfer and did appear in samples frozen with glycerol. The immediate transfer and frozen slough treatments were the least similar from the previous generation, suggesting inherent variability when transferring communities between hosts. However, overall results for the mouse to mouse transplant experiments indicate microbiome communities can be reestablished for *in vivo* modeling following cryopreservation.

It was inferred from analysis of PMA-qPCR results that overall bacterial viability was not reduced as a result of the freezing process (Fig 2). Had freeze treatments posed a significant reduction on the viability of sampled microbiota, a demographic bottleneck for bacterial lineages would have resulted in a loss of microbial species in wound models, and no significant reduction in alpha diversity was observed across preservation strategies. However, a significant loss of diversity was recorded in the process of transferring wound communities from human to mouse indicating host-specific effects on species establishment. A related potential hypothesis when reestablishing polymicrobial communities is that the cryogenic process may select for specific lineages that are robust to freezing and thawing. Literature has suggested that the cell wall characteristics of Gram negative bacteria may reduce species viability after freezing [31]. In the current study, variability in colonization success could not be attributed to Gram stain or aerotolerance, and it is possible that biofilm material provides a protective effect. A refined assessment of cryogenic selection on bacterial lineages could be later achieved by PMA-Seq [35].

Finding mice microbiota to be different from their respective patient microbiota is unsurprising given the change in host context from human to mouse (Fig 3). However, patient of origin consistently explained more variation than preservation type and mice of same patient origin were most similar to each other. These findings indicate colonization by slough is achieved, however the colonization process may be expected to vary among replicates, and commonly only a few species detected in the patient wounds were later detected in the mouse wounds. The relatively small sample size of patients included in this study did not allow for a complete sampling of microbes routinely observed in the wound clinic, but microbes observed in the current study are commonly reported [5]. *Staphylococcus aureus* appeared to dominate most mouse wounds, even when only present in relatively minute proportions of the prior patient community (Fig 3, S1 Table). This is consistent with prior work which saw *S*. *aureus* average relative abundance more than doubled from patient to mice [4]. Differences in observed wound microbiota could have also been due to wound heterogeneity within biofilm material, however inoculum load explained a significant portion of variation in community similarity (Fig 4A). Furthermore, patient of origin was also found to significantly explain variation in bacterial load among mice, indicating a positive relationship between the bacterial load in patient isolate and the load after colonization in mice.

Bacterial load and the prior relative abundance of individual species were positively associated with colonization success (Figs 4 and 5). Bacterial load has been previously documented to impact viability after freezing [31] and infection into animal models [23] with monoclonal samples. Results presented here show that bacterial load can also serve as a predictor of colonization success in polyclonal samples, as increased bacterial bioburden yielded model wounds that were more similar to the original patient while samples with low bioburden yielded less similar infections (Fig 4A). However, the relationship between bioburden and wound similarity is related to the observation that increased bioburden was negatively associated with alpha diversity in patient isolate. This pattern may be driven by species interactions that limit absolute abundances in increasingly complex communities (Fig 4B). Furthermore, a similar pattern was recently documented in polymicrobial cystic fibrosis sputum samples where infectious community types were found to have differential bioburden [41]; here the association between bioburden and biodiversity appears novel in the context of wound infection.

The prior relative abundance in wound inoculate was also found to positively relate to colonization success of individual bacterial species, where species in higher relative abundance were more likely to be detected in the mouse and, of species detected in mice, higher abundance was also found to influence the number of replicate mice in which a species was detected (Fig 5). Thus, successful colonization of a given species from patient to mouse was density dependent. However, an exception to this trend was *S*. *aureus* being able to persist and dominate community composition though being detected at low abundances in prior patient samples (Fig 3A, S1 Table). Furthermore, the observed success of *S*. *aureus* and *A*. *baumannii* likely reflects a bias for organisms capable of more rapid reproduction, especially in patient to model animal transfer. Overall, community similarity between patient and wound model appears influenced by the presence of fast-growing species, bacterial load, and prior relative abundance.

While a simplified goal of infecting mice with patient slough may be to re-create *in vivo* communities, prior investigation has shown that chronic wound microbiome communities are intrinsically variable through time [17, 18]. Our results clearly demonstrate that transplantation between wounds contributes additional variation, which may be an artifact of three related technical considerations. First, transplanting communities comes with a change in bacterial substrate. In addition to the obvious human to mouse differences mentioned above, physiological dynamics related to wound healing are likely to play a role in species establishment, for which a relatively acute wound and a wound having stalled in healing would hypothetically differ in niche availability [42, 43]. Relevant to this study, patients here received treatment in a wound present for at least 30 days, while the mice were sampled at four days post-wounding. Indeed, these effects were reduced from mouse to mouse transplant and more successful microbiome transfers may be achieved by only transferring between same-species hosts. Second, environmental filters that were present in the patient wound were not present in the mouse wound. Patients receiving treatment were also receiving antibiotics, which is known to act as a selective force on microbial communities. The third point relates to community succession since multi-species biofilm development has been previously noted to be dependent on the initial adherence of primary colonizers, who then recruit other species that benefit from positive interspecies dynamics [44]. Results suggest that the well-documented pathogen, *S*. *aureus* [4, 5, 17, 18], may act in a similar capacity in chronic wounds, but is also capable of dominating community structure at later time points [18]. Thus, the proportional re-establishment of complex wound communities in mouse models is expected to be influenced by the processes described above.

In conclusion, cryogenic preservation methods evaluated were overall not found to significantly influence community composition and the resulting communities were modeled after their previous community assemblage, though to varying degrees. Change induced in the transplanted microbiota was most significantly an effect of the transplantation process itself, and not cryogenic preservation. However, improved *in vivo* modeling via slough transfer may be further developed by recapitulating patient comorbidities such as diabetes in mice, administering therapeutics to mimic the selective environment of therapy or by repeated inoculation of wounds. A final implication of this study is that microbiome communities can be split into biological replicates and moved from mouse to mouse in a semi-controlled manner. The practicality of this method would then allow for studying polymicrobial communities in response to different environmental stressors through time, thereby providing a method to separate intrinsic microbiome variation from extrinsic treatment effects. Overall, cryogenic preservation and biofilm archives provide opportunities to enhance the study of polymicrobial infection dynamics.

## Supporting information

S1 Fig Rarefaction survey of wound microbiomes. (PNG)

S1 Table Species observed in patient wounds summarized by patient of origin, subsequent detection, relative abundance in patient and phenotypic characteristics. (PDF)
