# Supplementary figures and images for: Chronic wound microbiome colonization on mouse model following cryogenic preservation
Source: PLoS One. 2019 Aug 23;14(8):e0221565. doi: 10.1371/journal.pone.0221565 (PMC6707584; doi:10.1371/journal.pone.0221565)

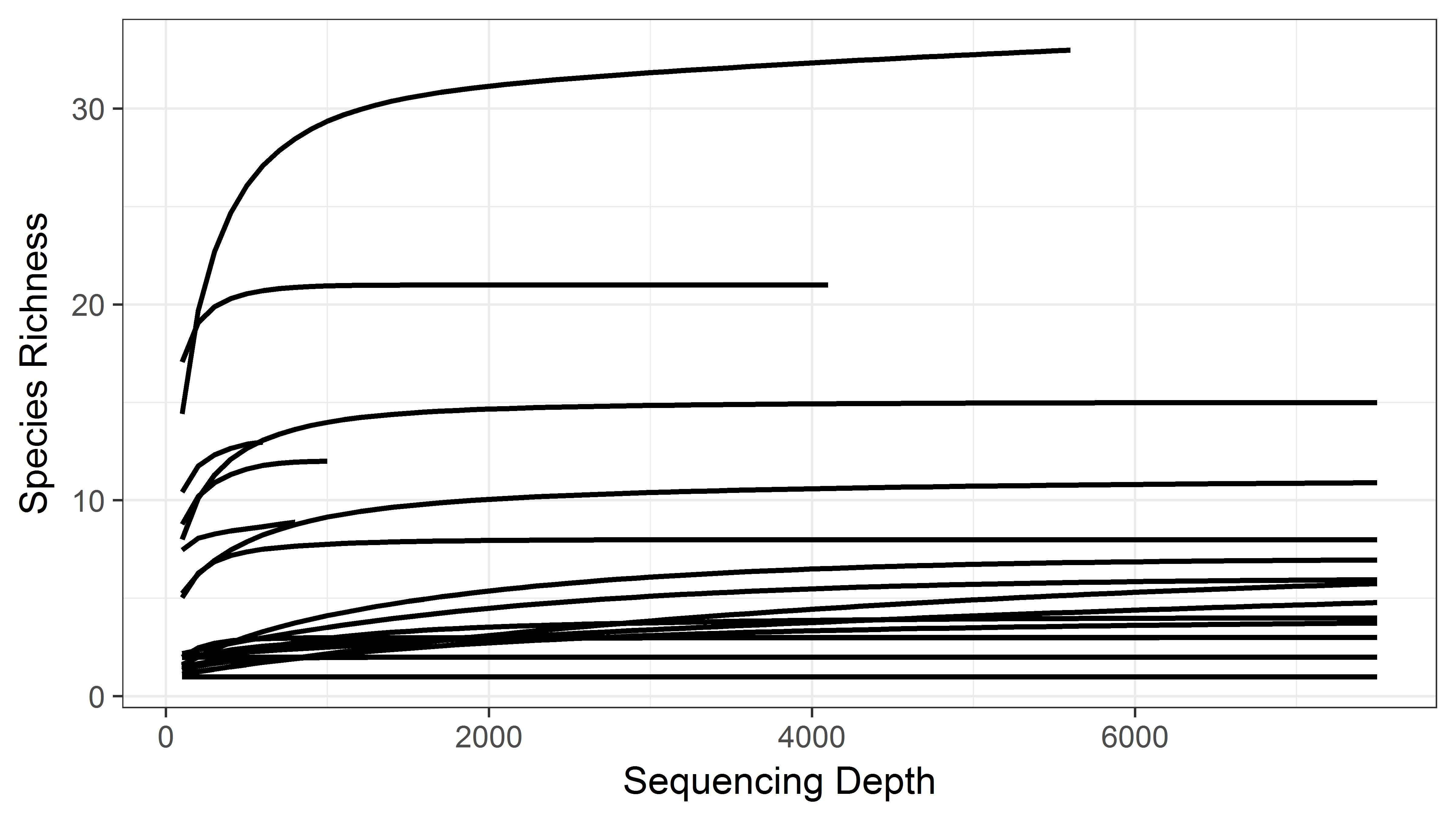

Supplement: S1 Fig — (PNG) [file pone.0221565.s001.png]
