# Supplementary material for: Chronic wound microbiome colonization on mouse model following cryogenic preservation
Source: PLoS One. 2019 Aug 23;14(8):e0221565. doi: 10.1371/journal.pone.0221565 (PMC6707584; doi:10.1371/journal.pone.0221565)
Supplement: S1 Table — (PDF) [file pone.0221565.s002.pdf]

Table S1. Species observed in patient wounds summarized by patient of origin, subsequent detection, relative abundance in patient and phenotypic characteristics.

Species	Patient	Detection	Relative Abundance	Gram	Aerotolerance
Acinetobacter baumannii	3	Detected	92.2340%	-	Aerobe
Acinetobacter baumannii	4	Detected	0.0003%	-	Aerobe
Acinetobacter baumannii	5	Detected	0.0009%	-	Aerobe
Anaerococcus lactolyticus	4	Detected	0.0156%	+	Anaerobe
Anaerococcus lactolyticus	5	Detected	0.1350%	+	Anaerobe
Anaerococcus prevotii	4	Detected	0.0013%	+	Anaerobe
Anaerococcus vaginalis	4	Detected	4.1945%	+	Anaerobe
Corynebacterium striatum	1	Detected	99.2924%	+	Facultative
Corynebacterium striatum	4	Detected	31.6827%	+	Facultative
Corynebacterium striatum	5	Detected	1.3811%	+	Facultative
Corynebacterium tuberculostearicum	2	Detected	1.6125%	+	Facultative
Helcococcus sueciensis	4	Detected	0.0005%	+	Facultative
Porphyromonas somerae	4	Detected	2.0023%	-	Anaerobe
Pseudomonas aeruginosa	4	Detected	0.0007%	-	Facultative
Staphylococcus aureus	1	Detected	0.0003%	+	Facultative
Staphylococcus aureus	4	Detected	0.1089%	+	Facultative
Staphylococcus aureus	5	Detected	0.0021%	+	Facultative
Turicella otitidis	2	Detected	0.2268%	+	Aerobe
Acinetobacter haemolyticus	5	Undetected	0.1731%	-	Aerobe
Anaerococcus prevotii	5	Undetected	0.0824%	+	Anaerobe
Anaerococcus vaginalis	5	Undetected	0.1813%	+	Anaerobe
Brevundimonas diminuta	2	Undetected	5.6689%	-	Aerobe
Brevundimonas vesicularis	5	Undetected	0.0360%	-	Aerobe
Burkholderia cepacia	5	Undetected	0.0109%	-	Aerobe
Finegoldia magna	4	Undetected	0.0116%	+	Anaerobe
Finegoldia magna	5	Undetected	0.0653%	+	Anaerobe
Helcococcus sueciensis	5	Undetected	0.1897%	+	Facultative
Peptoniphilus harei	4	Undetected	0.0052%	+	Anaerobe
Porphyromonas bennonis	4	Undetected	0.0021%	-	Anaerobe
Porphyromonas somerae	5	Undetected	0.2297%	-	Anaerobe
Pseudomonas stutzeri	5	Undetected	0.4033%	-	Aerobe
Staphylococcus epidermidis	1	Undetected	0.0002%	+	Facultative
Staphylococcus epidermidis	3	Undetected	0.1569%	+	Facultative
Staphylococcus epidermidis	5	Undetected	0.0115%	+	Facultative
Streptococcus agalactiae	5	Undetected	0.0315%	+	Facultative
